# One Method for Improving Overlay Accuracy Through Focus Control

**DOI:** 10.3390/mi17020207

**Published:** 2026-02-02

**Authors:** Yanping Lan, Jingchao Qi, Mengxi Gui

**Affiliations:** 1 Shanghai Yuwei Semiconductor Technology Co., Ltd., Shanghai 201203, China; 2 Changxin Memory Technologies, Hefei 230601, China

**Keywords:** IBO, overlay, dual-wavelength optical, focal plane measurement, mark alignment

## Abstract

Image-Based Overlay (IBO) equipment leverages optical reflection imaging principles, combined with focusing and alignment strategies to measure overlay marks. Among all measurement steps, the focal plane measurement of marks exerts the most critical impact on overlay accuracy, while the time consumed by focal plane detection directly determines the overall measurement throughput. To address the trade-off between accuracy and efficiency in advanced process nodes, this paper proposes an integrated optimization strategy encompassing optical hardware design and software algorithms. The hardware solution adopts a dual-wavelength, dual-detector architecture: optimal imaging wavelengths are selected independently for the previous-layer and current-layer marks, ensuring each layer achieves ideal imaging conditions without mutual interference. The software strategy employs a deep learning framework to simultaneously predict and adjust the horizontal position (alignment) and vertical defocus number of measured marks in real time with high precision, thereby securing the optimal imaging posture. By synergizing hardware-based optimal imaging conditions and software-based posture adjustment, this method effectively mitigates the impact of background noise and system aberrations, ultimately improving both the accuracy and efficiency of overlay measurement.

## 1. Introduction

Overlay accuracy, alongside resolution and throughput, stands as one of the three core performance indicators of lithography technology [1,2]. It directly dictates the electrical connectivity of multi-layer circuits and the final yield of semiconductor chips. With the advancement of process nodes into the 14 nm, 7 nm, and even 3 nm regimes, the critical dimension (CD) of devices has shrunk to single-digit nanometers, requiring overlay accuracy to be controlled below 2 nm (3σ) [3]. This places unprecedented demands on overlay metrology systems, which primarily consist of two technical routes: Image-Based Overlay (IBO) and Diffraction-Based Overlay (DBO) [4].

IBO equipment leverages optical reflection imaging principles to directly capture mark images, offering intuitive interpretation and robustness against certain process variations. In contrast, DBO relies on diffracted signals from periodic grating marks, extracting overlay information through spectral analysis. While DBO typically achieves higher precision and repeatability for certain film stacks due to its sensitivity to phase information, it often requires dedicated grating targets and is more susceptible to process-induced asymmetries and thin-film interference effects. Moreover, DBO systems may struggle with complex multi-layer stacks where multiple materials induce spectral crosstalk, limiting material versatility. IBO, with its direct imaging approach, offers greater flexibility in mark design and compatibility with diverse film systems, though traditionally at the cost of lower throughput and susceptibility to focus-induced errors.

The proposed method addresses key limitations of traditional IBO—namely, wavelength compatibility, throughput, and noise sensitivity—through an integrated hardware and software co-design. Compared to DBO, our approach retains IBO’s material versatility and adaptability to existing Box-in-Box marks while significantly closing the gap in precision and repeatability. By enabling dual-wavelength simultaneous imaging and deep learning-based joint alignment-focusing, our solution achieves sub-nanometer focal plane control and high throughput, making it competitive with DBO in advanced nodes, particularly in environments with complex film stacks and high process variability.

### 1.1. Related Work

Recent studies have attempted to improve IBO accuracy through optical optimization or machine learning. Multi-color per-layer imaging approaches have been proposed to minimize crosstalk in advanced film stacks, while ML-based residual reduction methods focus on TIS modeling [5]. However, these methods still rely on sequential focusing and alignment and typically use a single camera.

Deep learning has been applied to mark detection [6] and classification [7], but joint alignment–focus prediction remains unexplored. Moreover, prior multi-wavelength methods rely on sequential illumination rather than true parallel acquisition. The proposed method introduces simultaneous dual-wavelength acquisition with integrated deep-learning-based optimization, offering a new solution pathway.

### 1.2. Principles of IBO Measurement

IBO equipment operates on the principle of optical reflection: an optical system projects overlay marks (consisting of previous-layer and current-layer patterns) onto an image sensor (camera). Post-imaging, specialized software analyzes the centroid positions of the two layers of marks; the positional offset between these centroids is defined as the overlay error (Misregistration). Common overlay mark designs include Box-in-Box, Bar-in-Bar, and Advanced Imaging Metrology (AIM) marks [8], with Box-in-Box being the most widely used in mass production due to its simplicity and robustness.

**Figure 1:**
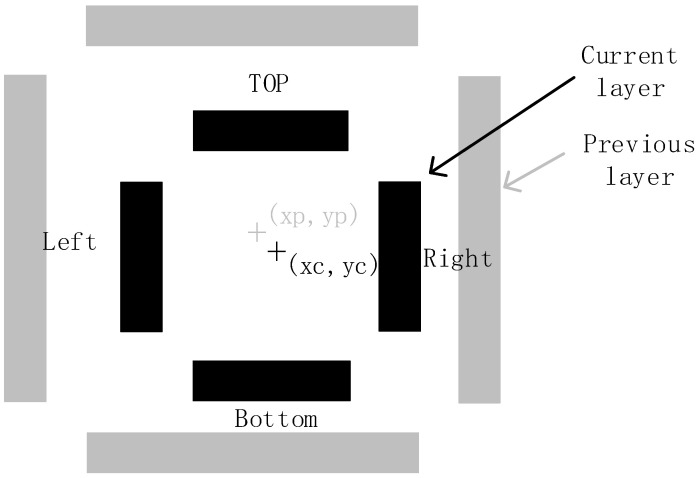
Bar-In-Bar overlay mark.

A typical Box-in-Box overlay mark is shown in Figure 1, consisting of two concentric rectangular frames. The gray outer frame represents the previous-layer mark, with its centroid at coordinates (xp, yp); the black inner frame represents the current-layer mark, with its centroid at (xc, yc). Each frame comprises four orthogonal bars: Top, Bottom, Left, and Right, all with identical length (20 μm) and width (1~2 μm) in this example. For horizontal (x-direction) overlay measurement, the Left and Right bars are used (y-direction serves as the non-measurement axis); for vertical (y-direction) measurement, the Top and Bottom bars are used (x-direction serves as the non-measurement axis). The overlay error is calculated as: Misregistration X = xc − xp (horizontal offset) and Misregistration Y = yc − yp (vertical offset).

**Figure 2:**
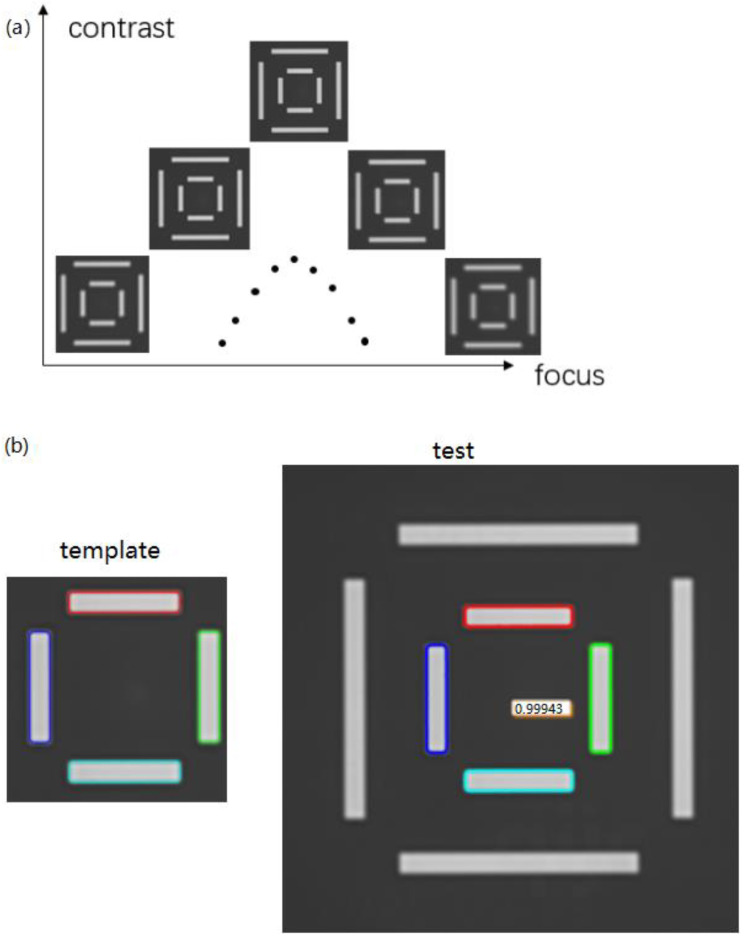
Contrast Calculated Focal Plane and Template Matching Alignment.

A flowchart illustrating the sequential focusing and alignment process in traditional IBO systems is illustrated in Figure 2. In Figure 2a, the x-axis denotes the focus position of the objective lens, scanned in discrete increments, while the y-axis represents the computed contrast of the alignment mark image. The nested square patterns illustrate the alignment mark at different focus positions, showing increasing sharpness as the lens approaches the optimal focal plane and decreasing sharpness as it moves away. The dotted curve plots contrast against focus position, forming a distinct peak; the apex of this peak identifies the optimal focal plane, where mark edge definition is maximized to enable precise overlay measurement. In Figure 2b, colors in the template and test images represent different alignment mark edges, used to determine horizontal position (x, y) via template matching.

The workflow starts with “Rough Focusing” (step 1), where the objective lens moves in 100 nm increments to capture a series of low-contrast images. Next, “Template Matching” (step 2) compares these images to a pre-stored mark template to determine the horizontal position (x, y) of the mark and center it in the FOV (reducing symmetric aberration effects). “Fine Focusing” (step 3) then uses a contrast calculation algorithm (e.g., Laplacian variance) to find the focal plane with the highest edge sharpness (defocus amount = 0). Finally, “Image Capture” (step 4) acquires the high-quality image for overlay analysis. The total time for steps 1–3 is ~50 ms, with fine focusing accounting for 60% of this duration.

### 1.3. Limitations of Traditional IBO Schemes

Traditional IBO systems adopt a single optical path, single-wavelength, and single-camera configuration, paired with a high-numerical-aperture (NA ≥ 0.7) achromatic objective lens to reduce chromatic aberration. However, this design faces three key challenges in advanced processes:

1. Wavelength Compatibility Issues: For complex film stacks (e.g., SiN hard masks, EUV photoresists), a single wavelength cannot simultaneously ensure clear imaging of both previous-layer and current-layer marks. For instance, visible light (405–633 nm) is blocked by SiN films (>200 nm thick), while near-infrared (NIR) light (900–1100 nm) struggles to resolve fine current-layer marks due to lower diffraction-limited resolution [9].
2. Low Throughput from Sequential Focusing/Alignment: To ensure optimal imaging, traditional schemes require sequential operations. This sequential workflow takes about 50 ms per mark, limiting the throughput to <30 wafers per hour for high-density sampling (200 marks per wafer) [2].
3. Background Noise Interference: During image acquisition, the sensor captures not only the signal from the mark but also background signals (e.g., scattered light from the wafer surface, reflections from underlayers). This reduces the signal-to-noise ratio (SNR) of mark edges, introducing measurement residuals of up to 0.3 nm [8].

To address these limitations, this paper proposes a dual-wavelength, dual-camera hardware architecture combined with a deep learning-based joint alignment-focusing algorithm. This integrated solution enables parallel imaging of previous and current layers, while simultaneously optimizing mark position and focal plane—thus improving both accuracy and throughput.

## 2. Dual-Wavelength Optical Scheme

The selection of the light source is pivotal to IBO performance, as wavelength directly impacts mark contrast, film penetration, and measurement repeatability. The evolution of IBO light sources and their corresponding performance metrics are summarized in Table 1. Over time, IBO light sources have evolved from white halogen lamps (broad spectrum, low intensity) to laser-driven light sources (LDLS) with color filters, and finally to wavelength tuners (continuous wavelength adjustment), as shown in Table 1. This evolution reflects the growing demand for wavelength flexibility in advanced processes.

### 2.1. Evolution of IBO Light Sources

**Figure 3:**
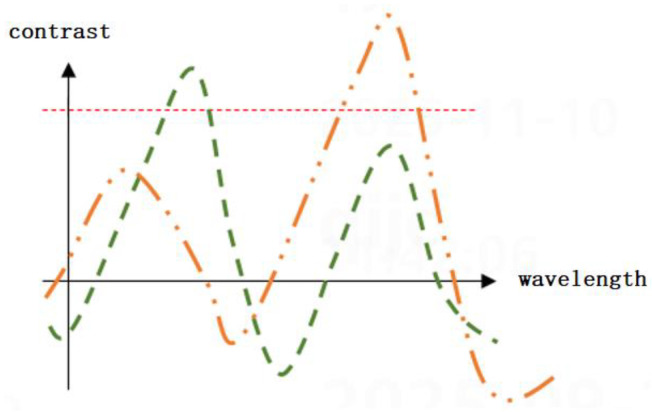
Contrast and Optimal Wavelengths for Two Layers.

For nodes < 14 nm, the complexity of film stacks (e.g., multi-layer SiN/SiO_2_, metal gates) makes it impossible to find a single wavelength that satisfies both “ previous-layer penetration” and “current-layer resolution” [9]. As shown in Figure 3, the previous-layer mark (under a 300 nm SiN film) achieves maximum contrast at 940 nm (NIR), while the current-layer mark (EUV photoresist) performs best at 405 nm (deep ultraviolet, DUV). A single wavelength (e.g., 633 nm) results in contrast values < 20 for both layers, leading to centroid extraction errors.

Table 1 illustrates the evolution of overlay metrology light source technologies and their corresponding TMU performance across different semiconductor process nodes. TMU (Total Measurement Uncertainty) is a key indicator evaluating the precision of overlay metrology systems, with smaller values representing higher measurement accuracy, where $TMU=\sqrt{{TIS}_{3\delta }^{2}+{TIS}_{Mean}^{2}+{Precision}^{2}}$.

Figure 3 shows the contrast (y-axis) vs. wavelength (x-axis) curves for previous-layer and current-layer marks. The orange dashed line represents the previous-layer mark (under a 300 nm SiN hard mask): contrast increases with wavelength, peaking at 940 nm due to enhanced penetration of NIR light through SiN. The green curve represents the current-layer mark (100 nm EUV photoresist): contrast peaks at 405 nm because DUV light provides higher diffraction-limited resolution for fine features. The red dashed horizontal line serves as a KPI threshold. Wavelength ranges where the curves exceed this threshold are deemed optimal, as the KPI meets or surpasses the required performance standard in these intervals. This validates the need for dual wavelengths to optimize each layer independently.

### 2.2. Dual-Wavelength, Dual-Camera Hardware Design

The dual-wavelength optical scheme retains the core reflection imaging principle of traditional IBO but modifies the hardware to enable parallel, layer-specific imaging. The key components include: (1) two independent LDLS light sources with wavelength tuners; (2) a dichroic prism (beam splitter); (3) two high-resolution cameras; and (4) a shared objective lens (to ensure coaxiality of the two optical paths).

**Figure 4:**
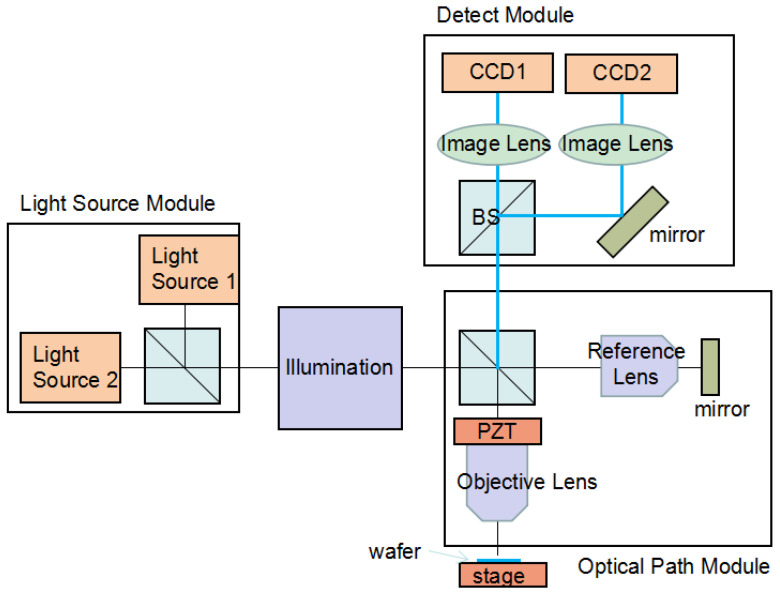
Dual-Wavelength Optical Path.

This optical scheme is a high-precision dual-wavelength, dual-camera optical system for semiconductor Overlay measurement, achieving high-precision alignment and measurement of wafers through multi-module collaboration. Figure 4 shows the Dual-Wavelength Optical Path. It mainly consists of three parts: light source module, illumination and optical path module, detection module.

1. Light Source Module: The light source module (Light Source Module) is equipped with two light sources (Light Source 1, Light Source 2) that can output light of two different wavelength bands (e.g., visible light & near-infrared, deep ultraviolet & infrared) to achieve “dual-wavelength” division of labor. One wavelength is used to penetrate multi-layer structures such as hard masks and photoresists on the wafer surface to identify lower-layer marks, while the other wavelength is used to enhance the edge contrast of upper-layer patterns for precise positioning of upper-layer marks. The two light sources are combined into a single beam via a beam-combining element before entering the illumination system.
2. Illumination and Optical Path Module: The illumination unit (Illumination) homogenizes and collimates the combined light to ensure light field uniformity. The light is split into a measurement optical path and a reference optical path. In the measurement optical path, the light is focused onto the wafer surface via an objective lens (Objective Lens). The wafer is placed on a high-precision wafer stage that enables nanometer-level motion control. After reflection by the wafer, the light carries structural information of the measured pattern and returns. In the reference optical path, the light forms reference light via a reference lens (Reference Lens) and a mirror (Mirror), which is used for subsequent interference or image comparison to eliminate systematic errors. Among them, a piezoelectric ceramic (PZT) can drive optical components to achieve nanometer-level displacement for precise focusing or phase modulation, further improving measurement accuracy.
3. Detection Module: The detection module (Detector Module) adopts a dual-camera (CCD1, CCD2) architecture. A beam splitter (BS) separates the dual-wavelength light carrying measured information, which is then focused via imaging lenses (Image Lens) and synchronously captured by two CCD cameras (i.e., the “Double Grab” function). The synchronization of the two cameras ensures that the upper and lower layer marks are imaged at the same time and in the same FOV, providing a foundation for accurate registration of subsequent Overlay algorithms. In addition, beam-splitting elements can adjust the beam-splitting ratio or imaging magnification of the optical path to adapt to the accuracy requirements of different measurement scenarios.

The advantages of this design:

1. Independent Wavelength Optimization: Each layer uses its optimal wavelength (e.g., 940 nm for previous-layer SiN penetration, 405 nm for current-layer resolution), maximizing mark contrast.
2. Parallel Imaging: Both layers are imaged simultaneously (Double Grab, DG) instead of sequentially, reducing per-mark measurement time.
3. Reduced Cross-Layer Interference: Since each camera only captures one wavelength, there is no signal crosstalk between previous and current layers—critical for thin-film stacks with high light scattering.

## 3. Alignment and Focusing Model Based on Deep Learning

While the dual-wavelength hardware improves imaging quality, optimal overlay accuracy still requires precise alignment (horizontal position) and focusing (vertical focal plane) of the mark. Traditional sequential methods (template matching + contrast calculation) are slow and prone to noise. To address this, we propose a multi-task deep learning model that simultaneously predicts the mark’s horizontal bounding box (alignment) and optimal focal plane (defocus amount) from a single image.

### 3.1. Model Inputs and Outputs

The model takes two types of inputs to account for both image information and system variability:

1. Image Input: A grayscale image of the overlay mark (resolution: 512 × 512 pixels; FOV: 10 μm × 10 μm), captured under either DUV or NIR light.
2. Optical Parameter Input: A K-dimensional vector of system parameters (K = 5 in this study) that affect imaging, including: wavelength (nm), light source intensity (mW), objective lens NA, camera exposure time (μs), and wafer film thickness (nm).

The model outputs two key results for overlay measurement:

3. Alignment Output: A bounding box (x, y, w, h) defining the mark’s position in the FOV (x, y = top-left corner coordinates; w, h = width/height of the box).
4. Focus Output: The optimal focal plane position (z, in nm), defined as the vertical distance between the objective lens and the mark surface that maximizes edge contrast.

### 3.2. Model Architecture

**Figure 5:**
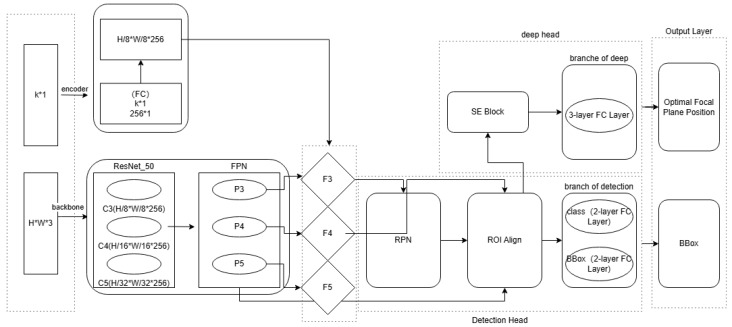
Schematic Diagram of the Deep Learning Model Structure.

The architecture adopts an end-to-end design, integrating image feature extraction, optical parameter encoding, feature fusion, and multi-task prediction (Figure 5).

Figure 5 Description: The model architecture consists of five core modules: (1) Backbone Network (Image Feature Extraction); (2) Optical Parameter Encoder; (3) Feature Fusion; (4) Multi-Task Head (Detection + Depth); and (5) Loss Function. Detailed breakdown:

1. Backbone Network (Image Feature Extraction): ResNet-101 is used to extract multi-scale image features, outputting three feature maps (C3: 64 × 64 × 256; C4: 32 × 32 × 512; C5: 16 × 16 × 1024) after 8×, 16×, and 32× downsampling. A Feature Pyramid Network (FPN) then generates P3–P7 feature maps (P3: 64 × 64 × 256; P7: 2 × 2 × 256) to capture both fine-grained (P3) and global (P7) features.
2. Optical Parameter Encoder: A 2-layer fully connected (FC) network processes the 5-dimensional optical parameters, outputting a 256-dimensional feature vector (h_opt) via ReLU activation. Optical parameters encoded into feature vectors compatible with image features.
3. Feature Fusion (Image Features and Optical Parameters): h_opt is spatially expanded to match the size of P3 (64 × 64 × 256) using bilinear interpolation, then fused with P3–P5 via element-wise addition (residual connection) to inject system parameter information into image features.
4. Multi Task Head contains detection head and depth head. Detection Head (Alignment) Follows the Faster R-CNN framework: a Region Proposal Network (RPN) generates anchor boxes on P3–P5; ROI Align extracts 7 × 7 × 256 features for each anchor; two FC layers predict class confidence (mark vs. background) and bounding box offsets. Depth Head (Focus) Uses a Squeeze-and-Excitation (SE) Block to weight ROI features (enhancing edge-related regions), followed by three FC layers to regress the focal plane z-value.
5. Loss Function: Total loss = λ_1_ × Detection Loss + λ_2_ × Depth Loss. λ_1_ = 1.0, λ_2_ = 0.5; hyper parameters tuned via cross-validation. The values of λ_1_ = 1.0 and λ_2_ = 0.5 in the loss function are based on 5-fold cross-validation: four combinations of λ_2_ = 0.3, 0.5, 0.7, and 1.0 were compared (with λ_1_ fixed at 1.0), and it was found that when λ_2_ = 0.5, the comprehensive error of alignment precision (IoU ± 0.3 pixel) and focusing accuracy (±8 nm) is minimized. Sensitivity analysis shows that when λ_2_ > 0.7, the weight of focusing loss is too high, leading to an increase in alignment bounding box offset (IoU drops to ±0.5 pixel); when λ_2_ < 0.3, the weight of alignment loss is too high, resulting in an increase in focusing accuracy is ±12 nm, neither of which can meet the requirements of advanced processes.

### 3.3. Loss Function Definition

To balance alignment accuracy and focal plane precision, the model uses a combined loss function. The detection loss includes classification loss (Focal Loss, to address class imbalance between mark and background) and bounding box regression loss (GIoU Loss, to improve localization accuracy).

Detection loss:

$$L_{\det}=L_{\mathrm{cls}}+L_{\mathrm{box}}$$

$$L_{\mathrm{cls}}=-\frac{1}{N_{\mathrm{pos}}}\sum_{i=1}^{N_{\mathrm{pos}}}\alpha {\left(1-\hat{p_{i}}\right)}^{\mathrm{\gamma log}}\left(\hat{p_{i}}\right)-\frac{1}{N_{\mathrm{neg}}}\sum_{j=1}^{N_{\mathrm{neg}}}\left(1-\alpha \right){\left(\hat{p_{j}}\right)}^{\mathrm{\gamma log}}\left(1-\hat{p_{j}}\right)\;(Focal\;Loss)$$

$$L_{\mathrm{box}}=1-GIoU\left(\hat{b},b\right)$$

Focal plane regression loss: The focal plane regression uses L1 Loss (robust to outliers) to minimize the difference between predicted $\hat{z}$ and ground-truth z focal plane positions.

$$L_{\mathrm{depth}}=\left|\hat{z}-z\right|$$

Total loss:

$$L_{\mathrm{total}}=\lambda_{1}L_{\det}+\lambda_{2}L_{\mathrm{depth}}\;(\lambda_{1},\lambda_{2}\;are\;hyperparameters)$$

### 3.4. Model Training and Validation

The model was trained on a dataset of 10,000 overlay mark images captured from a 7 nm DRAM wafer. Each sample included: (1) a 512 × 512 grayscale image; (2) 5-dimensional optical parameters; (3) ground-truth bounding box (labeled manually); (4) ground-truth focal plane (measured via high-precision interferometry). The dataset covered 50 different film stacks and 10 wavelength settings (405–940 nm).

Training was performed on an NVIDIA A100 GPU for 100 epochs, with a batch size of 32 and Adam optimizer (learning rate = 1 × 10^−4^). Validation on a holdout set (2000 samples) showed the following:

Alignment accuracy: Bounding box IoU (Intersection over Union) with ground truth is ±0.3 pixel.

Focus accuracy: Mean absolute error (MAE) between predicted and ground-truth is ±8 nm.

## 4. Experimental Results and Discussion

To validate the proposed method, experiments were conducted on a 7 nm DRAM wafer (200 overlay marks per wafer) using two setups: (1) Traditional IBO (single wavelength: 633 nm; sequential alignment/focusing); (2) Proposed IBO (dual wavelength: 405 nm + 940 nm; deep learning-based alignment/focusing). Key performance metrics—contrast, alignment/focus time, and focal plane repeatability—were compared.

### 4.1. Imaging Contrast Improvement

Contrast is the maximum value of the gradient signal. SNR (Signal-to-Noise Ratio) is calculated as the ratio of edge signal power to background noise power. The dual-wavelength scheme nearly doubled the contrast for both layers, attributed to: (1) 940 nm NIR light’s enhanced penetration through SiN (previous layer); (2) 405 nm DUV light’s high resolution for current-layer photoresist marks. Higher contrast reduces centroid extraction error—critical for sub-2 nm overlay accuracy.

The quantitative comparison of contrast performance between the two schemes is presented in Table 2. This table compares the performance of two schemes (traditional single-wavelength vs. proposed dual-wavelength) in terms of mark contrast and SNR. The dual-wavelength scheme also outperforms the traditional one (38.7 ± 2.3 vs. 15.2 ± 1.5), indicating better signal quality at mark edges. In summary, the dual-wavelength scheme significantly enhances both contrast (for both layers) and SNR compared to the traditional single-wavelength approach.

### 4.2. Throughput and Repeatability Improvement

The throughput and repeatability metrics of the proposed method versus the traditional scheme are presented in Table 3. The traditional scheme requires 50 ± 3 ms, while the proposed scheme reduces this to 12 ± 1 ms, achieving a 76% improvement in speed. The traditional scheme has a repeatability of 23 ± 2 nm, whereas the proposed scheme achieves 11 ± 1 nm, delivering a 52% improvement in positioning accuracy.

**Figure 6:**
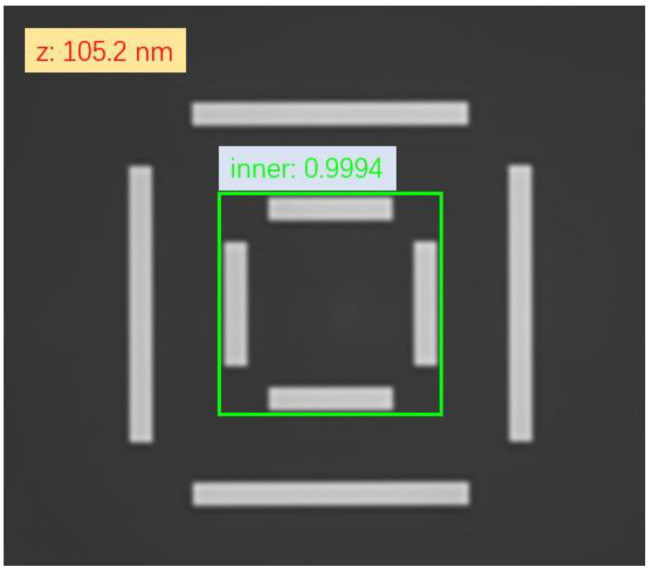
Focal Plane Prediction and Mark Position Prediction.

A sample output from the proposed deep learning model is illustrated in Figure 6. The left panel shows the input image. The green box is the model-predicted bounding box (IoU with ground truth = 0.9994); the red text shows the predicted focal plane z = 105.2 nm (ground truth = 100.0 nm, error = 5.2 nm). The right panel shows the focal plane repeatability: 3σ of 100 measurements on the same mark = 8.0 nm for the proposed model, compared to 12 nm for the traditional contrast method. The reduced scatter of the proposed model (blue dots) indicates better resistance to background noise.

## 5. Conclusions

This paper presents an integrated solution for improving IBO overlay accuracy, combining a dual-wavelength, dual-camera hardware design and a multi-task deep learning algorithm. The dual-wavelength architecture enables independent optimization of imaging conditions for previous-layer and current-layer marks, doubling mark contrast and SNR compared to traditional single-wavelength schemes. The deep learning model simultaneously predicts mark alignment and focal plane from a single image, reducing alignment/focus time from 50 ms to 12 ms (76% improvement) and improving focal plane repeatability by 52%. Experimental results on a 7 nm DRAM wafer show the proposed scheme achieves meeting the accuracy requirements.

Future work will focus on the following: (1) expanding the model to support EUV wavelengths for next-generation lithography; (2) integrating real-time film thickness estimation into the deep learning framework to further reduce calibration time; (3) miniaturizing the dual-wavelength optical system for in situ overlay measurement (embedded in lithography scanners).

## Figures and Tables

**Table 1:** Light Source Evolution and Corresponding TMU.

Process Node	Light Source	TMU (nm)	Key Limitation
>28 nm	White Halogen Lamp	0.75	Poor contrast for thin films; high noise
28~14 nm	LDLS + Color Filters	0.43	Limited wavelength options; cross-layer interference
<14 nm	LDLS + Wavelength Tuner	0.31	Single wavelength cannot optimize both layers

**Table 2:** Comparison of Mark Contrast Between Single-Wavelength and Dual-Wavelength Schemes.

Scheme	Previous-Layer Contrast (Outer Frame)	Current-Layer Contrast (Inner Frame)	SNR of Mark Edges
Traditional (633 nm)	18.3 ± 2.1	12.5 ± 1.8	15.2 ± 1.5
Proposed (Dual-WL)	35.3 ± 2.4	41.2 ± 2.7	38.7 ± 2.3

**Table 3:** Comparison of Throughput and Focal Plane Repeatability.

Metric	Traditional Scheme	Proposed Scheme	Improvement Percentage
Alignment + Focus Time per Mark	50 ± 3 ms	12 ± 1 ms	76%
Focal Plane Repeatability (3σ)	23 ± 2 nm	11 ± 1 nm	52%

## Data Availability

The original contributions presented in this study are included in the article. Further inquiries can be directed to the corresponding author.
